# Combination therapy of zinc and trimethoprim inhibits infection of influenza A virus in chick embryo

**DOI:** 10.1186/s12985-021-01585-1

**Published:** 2021-06-03

**Authors:** Magdi H. El Habbal

**Affiliations:** Mastropix Health Care, 35 Mew Walk, North Ferriby, HU14 3AH UK

**Keywords:** Chick embryo, Influenza virus, Trimethoprim, Zinc, Hemagglutination

## Abstract

**Background:**

Respiratory RNA viruses including influenza virus have been a cause of health and economic hardships. These viruses depend on its host for replication and infection. Influenza virus infection is lethal to the chick embryo. We examined whether a combination of trimethoprim and zinc (Tri-Z), that acts on the host, can reduce the lethal effect of influenza A virus in chick embryo model.

**Method:**

Influenza virus was isolated from patients and propagated in eggs. We determined viral load that infects 50% of eggs (50% egg lethal dose, ELD_50_). We introduced 10 ELD_50_ into embryonated eggs and repeated the experiments using 100 ELD_50_. A mixture of zinc oxide (Zn) and trimethoprim (TMP) (weight/weight ratios ranged from 0.01 to 0.3, Zn/TMP with increment of 0.1) was tested for embryo survival of the infection (n = 12 per ratio, in triplicates). Embryo survival was determined by candling eggs daily for 7 days. Controls of Zn, TMP, saline or convalescent serum were conducted in parallel. The effect of Tri-Z on virus binding to its cell surface receptor was evaluated in a hemagglutination inhibition (HAI) assay using chicken red cells. Tri-Z was prepared to concentration of 10 mg TMP and 1.8 mg Zn per ml, then serial dilutions were made. HAI effect was expressed as scores where ++++ = no effect; 0 = complete HAI effect.

**Results:**

TMP, Zn or saline separately had no effect on embryo survival, none of the embryos survived influenza virus infection. All embryos treated with convalescent serum survived. Tri-Z, at ratio range of 0.15–0.2 (optimal ratio of 0.18) Zn/TMP, enabled embryos to survive influenza virus despite increasing viral load (> 80% survival at optimal ratio). At concentration of 15 µg/ml of optimal ratio, Tri-Z had total HAI effect (scored 0). However, at clinical concentration of 5 µg/ml, Tri-Z had partial HAI effect (+ +).

**Conclusion:**

Acting on host cells, Tri-Z at optimal ratio can reduce the lethal effect of influenza A virus in chick embryo. Tri-Z has HAI effect. These findings suggest that combination of trimethoprim and zinc at optimal ratio can be provided as treatment for influenza and possibly other respiratory RNA viruses infection in man.

## Introduction

Respiratory RNA viruses are continuing to challenge man with epidemics and devastating pandemics. RNA and DNA synthesis require polymerase enzymes. While DNA polymerases have read-proof abilities with correction facilities, RNA polymerases do not have such qualities whereby errors, i.e. mutations, occur [1]. Some of these mutations may allow RNA virus to cross species like SARS and COVID-19 whereby no vaccination existed. Mutations of influenza virus continue to cause yearly epidemics and, in 1918 and 2009, instigated pandemics [2].

Influenza virus can adapt to species specific haemagglutinin biding receptors on cell surface and then cross the species [3]. Crossing species is a threat to national and international public health with devastating health and socioeconomics burdens [4]. In the wrong hands, a technology involving respiratory RNA viruses may become a threat to national security. Public health education, vaccination and drug treatment are strategies whereby national security against threats of respiratory RNA virus pandemics is ensured.

In anticipation of the threats, we embarked on development on an anti-viral. We identified trimethoprim (TMP) and zinc (Zn) as potential candidates for treatment for RNA virus. RNA virus requires host cell wall receptors to infect and its nucleus to replicate [2]. TMP inhibits folate dehydrogenase where-by it affects nuclear DNA with deleterious effect on the cell and its wall [5]. In man, the effect is transient and mostly subclinical [6]. Zinc ions were demonstrated to inhibit RNA virus, rhinovirus [7]. When combined at a ratio TMP and Zn may treat respiratory RNA virus infections [8].

There are several respiratory RNA viruses including influenza virus, Coronavirus, Respiratory Syncytial virus and rhinovirus [9]. To infect, these viruses require attaching to the host cell surface receptors, internalization and replication using the host nucleus and intracellular organelles [2, 8, 9]. We used influenza virus as a model because it shares similar mechanisms for infection, replication and pathogenesis with that of the respiratory RNA viruses [3, 9].

Studies of influenza virus are usually carried out in ferret and/or mouse models. However, ferrets and mice are more susceptible to zinc toxicity than other species such as the chick embryo [10, 11]. The chick requires zinc for normal development and can tolerate high intake of zinc supplied in appropriate diet [12, 13]. Influenza virus infection is lethal to the chick embryo. Chick embryo is easy to access as a culture medium, highly susceptible to influenza virus, of low cost and was shown to be a reliable model for testing anti-influenza drugs [14, 15]. In this study we examined the use of combination of TMP and Zn on chick embryo survival to influenza infection.

## Methods

We isolated influenza virus from positive swabs from patients during influenza season and propagated it in white leg horn embryonate eggs. Virus detection was carried out using florescence immune assay (FIA) and its subtypes identified by using PCR (RT-PCR, TaqMan, Thermofisher Scientific) as previously described [16, 17].

### Virus isolation

Virus was isolated from positive patient’s swabs during influenza season. Virus strain identification was conducted as previously described [16–18]. We identified the strain H3N2 [A/England/215/2011 (H3N2) like-virus].

We used embryonated eggs as described by and according to WHO guidance for virus isolation and detection [19, 20]. Briefly, eggs were incubated at 33–35 ℃ and 70% humidity with air sac facing upward (to allow embryo development at the air sac position for ease of visualization). The choice of 33–35 ℃ is to allow optimal viral multiplications [21]. Eggs were candled daily to check for embryo growth. Before inoculation, the stage of development was determined by direct inspection through a window in the egg shell using Olympus dissecting microscope. Eggs that did not show live embryos at matching stage of development were removed and discarded. The stage of development was determined according to Hamburger and Hamilton [22]. Thus, embryos were tested at the same stage of development. On day 8 of incubation (Hamburger and Hamilton stage 34) eggs were used for virus isolation.

Influenza positive material (0.2 ml) was mixed with broad spectrum antibiotic. The material was injected via the window using 22-gauge needle into amniotic and allantoic space. The window was sealed using bees wax. Eggs were candled daily to detect embryos survival. At day 11 of incubations, eggs were removed from incubator and placed in fridge at 4 ℃ for 1 h to reduce the likelihood of bleeding from allantoic membrane. The presence of the virus was determined by hem-agglutination (HA) test [23, 24].

We repeated the passage in chick embryos 3 times. From the last passage, allantoic fluids were collected to make a stock of the virus. The stock was tested for presence of the virus using standardized HA technique. Aliquots were made and stored in -70 ℃ till used.

### Determination of 50% egg infective dose (EID_50_)

It was made by inoculating 0.1 ml of serial tenfold dilutions of virus. Eggs were incubated at 36 °C and candled daily to detect embryo survival. On day 8 on incubation, we injected 0.1 ml of the virus into the allantoic cavity. The eggs were returned to the incubator for further 48 h at 36 °C. Eggs were opened to determine embryo survival or death. The allantoic fluid from each egg, was tested for the virus by HA test. We used 12 eggs per dilution and repeated the experiments 3 times. The dilution that caused 50% death of embryos (egg infectious doses, EID_50_) was determined using survival and death as described by Reed and Muench [25]. We calculated the dilution that contain 100 EID_50_ according to the equation: Log dilution of virus suspension containing 100 EID_50_ = Log EID_50_ + 2.

### Determining effect of Tri-Z

**Fig. 1:**
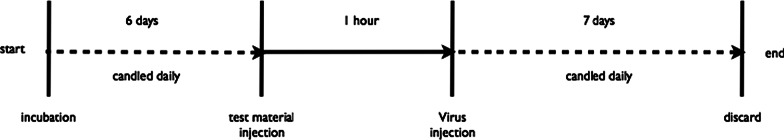
Schematic diagram showing study protocol. Test material were injected 6 days after start of egg incubation, Hamburger and Hamilton stage 29. An hour later, the virus was injected into the allantoic space. Eggs were returned to the incubator, between injections. It was candled daily for 7 days afterward to determine embryo survival, then discarded

Summary of the study protocol and groups are shown in Fig. 1 and Table 1, respectively.

**Table 1:** It shows study groups

Group	Virus load	Treatment	Number of embryos
group 1	10 EID_50_	Tri-Z: at Zn/TMP ratio 0.01 to 0.3, increment of 0.01 (30 ratios)	12 per ratio
group 2	100 EID_50_	Tri-Z: at Zn/TMP ratio 0.01 to 0.3, increment of 0.01 (30 ratios)	12 per ratio
C 1	10 EID_50_	.5 ml NS	12
C 2	10 EID_50_	Zn: 0.05 to 1.5 mg with 0.05 mg increments (30 concentrations)	12 per concentration
C 3	10 EID_50_	TMP: 5 mg	12
C 4	10 EID_50_	0.2 ml of convalescent serum	12

We carried experiments examining effect of a mixture of trimethoprim and zinc influenza virus. We examined two virus loads 10 EID_50_ and 100 EID_50_, in 6 day old chick embryos, group 1 and group 2. This was compared to control groups (C 1- C 4). Experiments were repeated in triplicates. EID_50_ = egg infective dose 50 i.e. virus load that cause 50% mortality of the embryo, C = control, NS = normal saline, TMP = trimethoprim, Zn = zinc, Tri-Z: mixture of trimethoprim and zinc oxide

The effect of Tri-Z on influenza virus was tested using embryo survival as measure of effectiveness. For these experiments, we used eggs at day 6 of incubation (Hamburger and Hamilton stage 29) because it is a vulnerable (interferon deficient) period for the embryo [26]. The outcome of treatment is measured by percentage of survival (effective) or death (ineffective) of the embryos 7 days after inoculation.

Embryo survival was determined as heart is beating, and embryo is moving. Death of the embryo was recorded as being the breakdown of the visible blood vessel structures of the chorioallantoic membrane, the appearance of disseminated coagulation, blood leakage into the yolk or the allantoic fluid areas, the absence of heart beating, absence of movement of the embryo, and extensive autolysis [19].

All experiments were repeated three times to ensure reproducibility.

We made suspensions from mixture of TMP and Zn (Tri-Z). The Zn/TMP ratio ranged from 0.01 to 0.3 with increment of 0.01. TMP powder (Medex, UK) was suspended in sterile water at concentration of 10 mg/ ml. We added Zn oxide powder (Medex, UK) to generate the desired mixture ratio. We made fresh suspension/s prior to each experiment. It was shaken to allow for homogeneous distribution of the compounds. We tested 30 ratios with 12 eggs in each ratio group.

### Study groups

On day 6 of egg incubation, the combined suspension was injected (0.5 ml) into the air sac using plastic syringe through an 18-gauge needle. An hour later, 10 EID_50_ was injected into the allantoic space as close as possible to the embryo (group 1). Eggs were returned to the incubator and candled daily for 7 days to determine embryo survival.

To evaluate the effect of increasing viral load, we repeated the same experiments but using 100EID_50_ virus suspension, (group 2).

### Controls

To verify the effects of Tri-Z on influenza virus, we carried out similar experiments in controls groups, at Hamilton and Hamburger stage 29 (day 6 of incubation). In each experiment, 12 fertilized eggs were used. We followed the study protocol but the test material varied based on the control group.

*First control* (C1): The test material was an increasing dose of Zn (in 0.5 ml saline, to match the amount of Zn in the respective ratio but without TMP) was injected into eggs in similar fashion.

*Second control* (C2): we used TMP only at a dose of 5 mg in 0.5 ml saline.

*Third control* (C3): we injected 0.5 ml of normal saline into the eggs.

*Fourth control* (C4): To ensure that death of embryo occurred from viral infection, virus infected eggs (10 EID_50_) were incubated with and without neutralizing antibodies using periodate treated convalescent serum (0.5 ml) from the patient as previously described [24].

### Hemagglutination (HA)

As previously described [23, 24], hem-agglutinin titrations were carried out by adding 0.2 ml of a 0.5% suspension of washed fowl erythrocytes (RBCs) to 0.2 ml of each serial two-fold dilution of test material. The tests were read after 45 min at room temperature. The end point was the highest dilution showing compete HA which contains 1 HA unit (HAU)/0.05 ml.

### Hemagglutination inhibition (HAI)

We carried out HAI as previously described [24] with Tri-Z as the test material. Tri-Z was prepared to concentration of 10 mg TMP and 1.8 mg Zn per ml, then serial dilutions were made. We added equal volumes of Tri-Z to that of 4 HAU of virus. Then, fowl RBCs were added. The final mixture was incubated for 30 min at room temperature before reading [23]. To verify that the effect of Tri-Z is on the cell and not the virus, we repeated the same experiment except that the RBCs were incubated with Tri-Z for 30 min, and then washed in PBS prior to addition to the virus. Results were expressed as previously described, where no HA = 0, some HA = +, partial HA = ++ , more than partial HA = +++, complete HA = ++++ [23, 24].

### Statistics

We used the number of embryos surviving at day 7 after infection as a measure of the outcome of treatment. Data were tested for normality of distribution, and then parametric or non-parametric tests were applied accordingly. If data were normally distributed, we applied paired test to determine the significance of change in ratio on the survival of embryos. Where data were abnormally distributed, we applied Mann–Whitney test. To determine the significance of difference in survival with change in ratio we applied repeated analysis of variance. Chi square test was applied to identify the most significant ratio at which highest survival was achieved. Significance value was set at p < 0.05 [27, 28].

## Results

The virus isolated from patients was influenza virus H3N2 [A/England/215/2011(H3N2) like-virus infection].

**Fig. 2:**
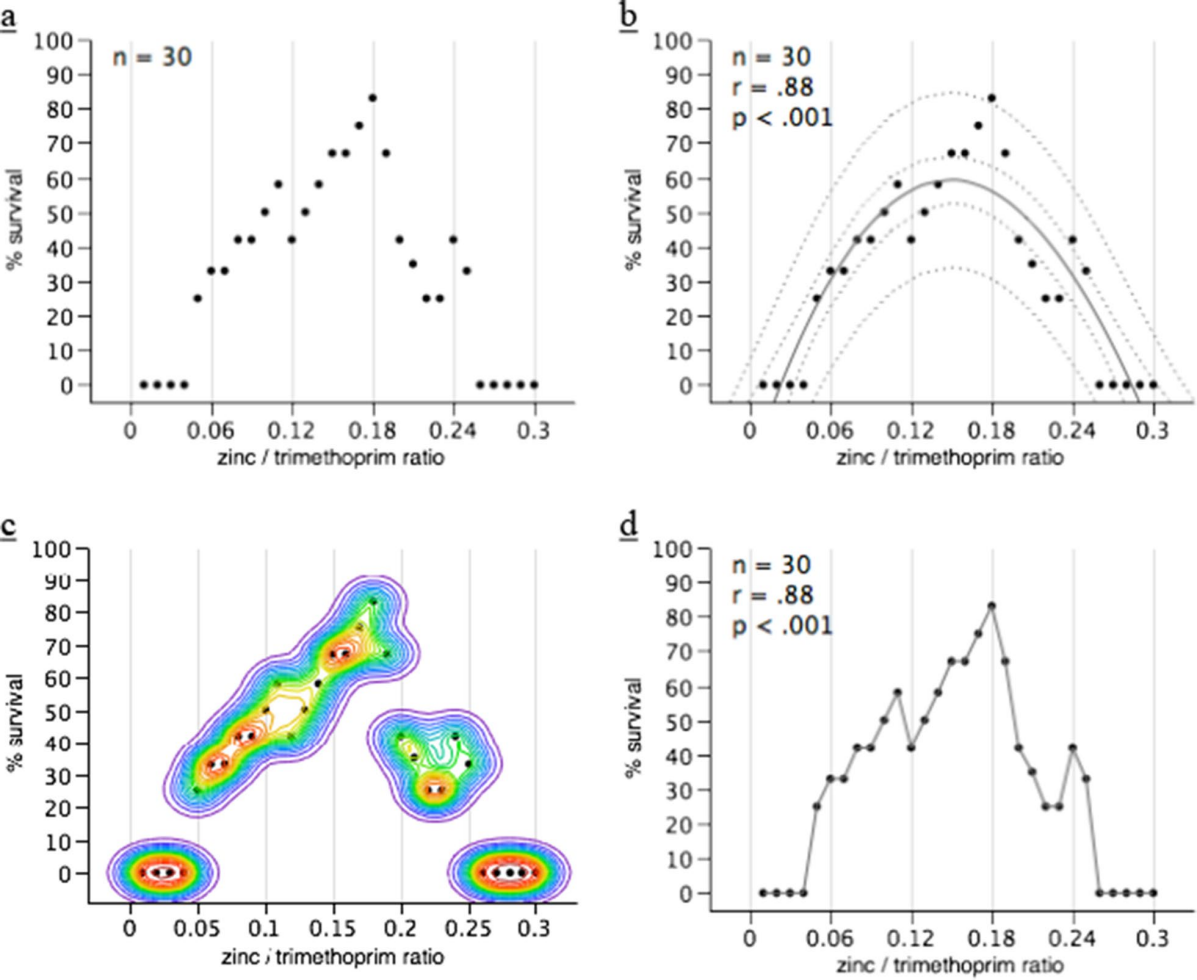
To determine the ratio of Zn to TMP that is most effective in treating viral influenza infection (10 EID_50_). Fertilised chicken eggs (n = 12) were infected with data shown with solid line connecting each data point. From (**a**) and (**d**) it seemed that the peak of survivors reached at Zn/TMP ratio of 0.18. However by analysing the data closely in (**b**) and (**c**) it appeared that the reliable Zn/TMP ratio is 0.15. (**a**) Data are displayed as solid points. (**b**) Polynomial equation (Y = 65.603867 − 41.570169 × X − 3474.1479 × (X − 0.155)^2^ + 1821.6868 × (X − 0.155)^3^) was applied and mean (solid line) 1 and 2 standard deviations (dotted lines) were plotted. **c** Area analysis with colour variation whereby orange-red represent consistent data. **d** same plot as a with data points joined by solid lines

**Fig. 3:**
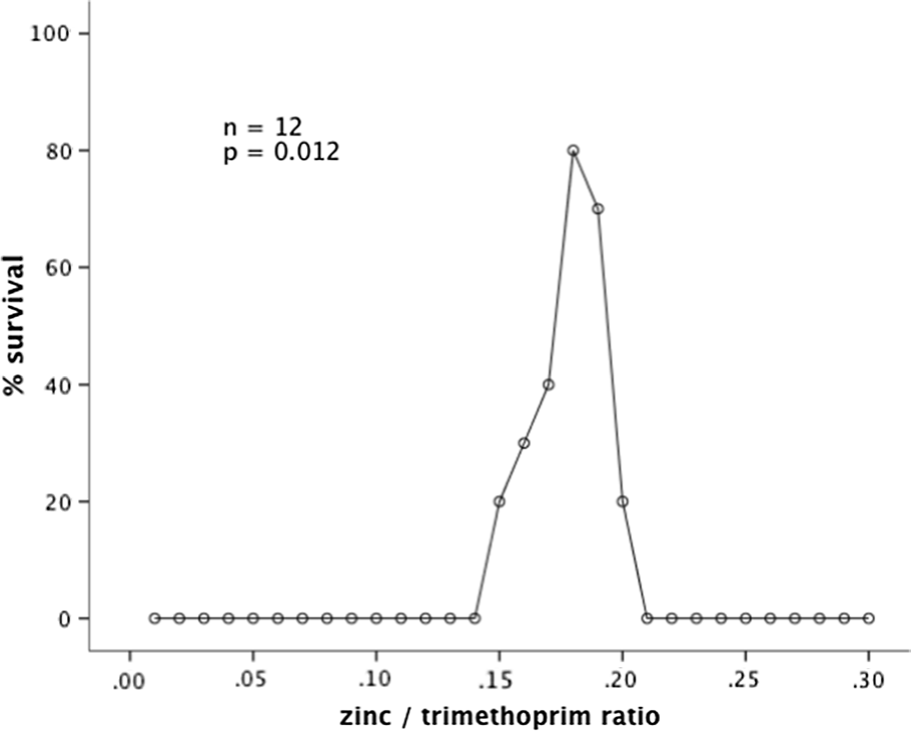
It shows the effect of high viral load (100 EID_50_) on chick embryo survival. We used the same ratios and doses as outlined in methods. Embryo survival was maximum at 0.18 ratio of Zn to TMP

**Fig. 4:**
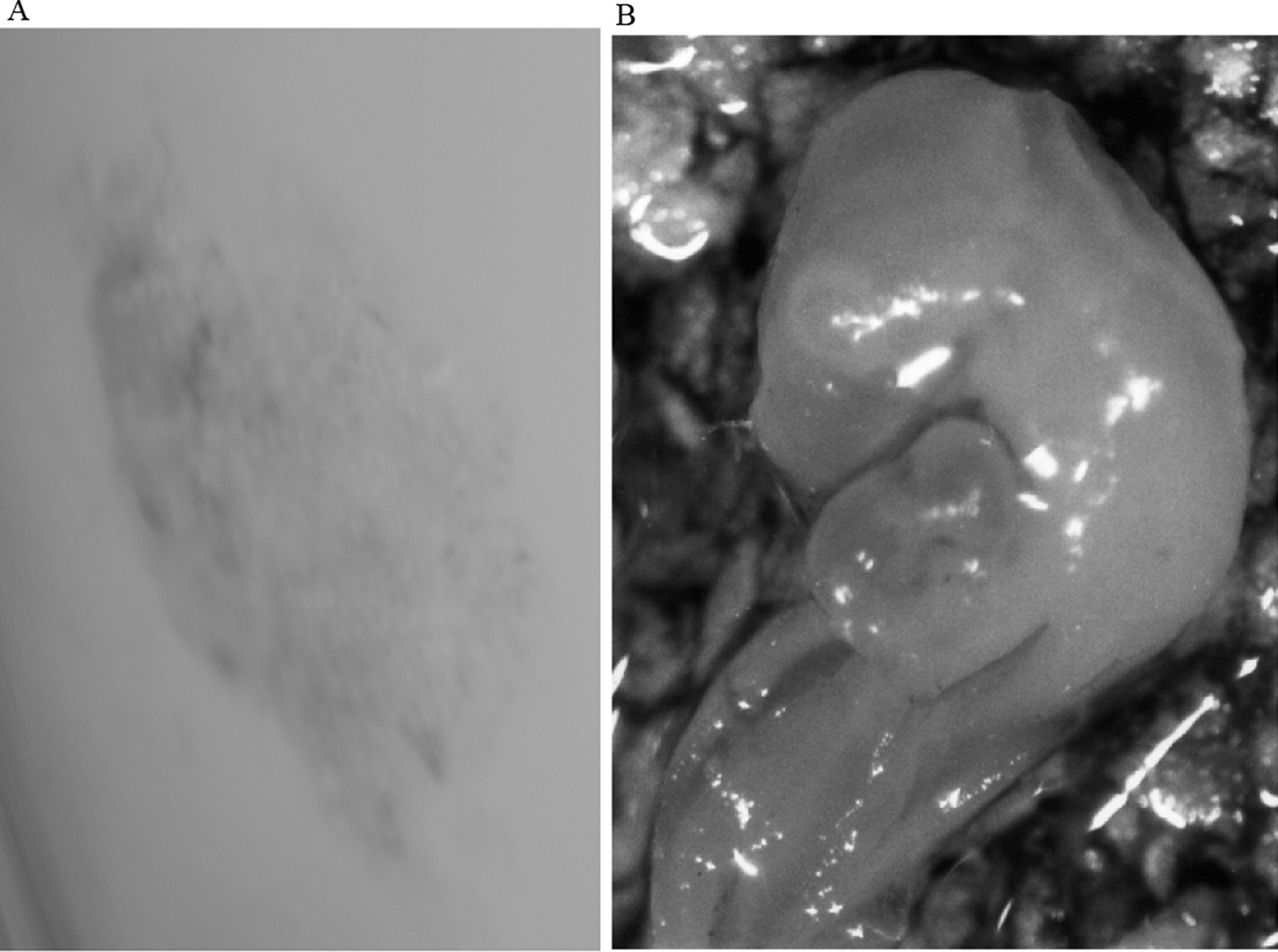
The effect of virus infection at 6 days of incubation on check embryo survival. **a** shows complete disintegration of the embryo and failure to grow compared to that treated with Tri-Z (**b**)

At viral load of 10EID_50_, there was no embryo survival at ratios 0.01 to 0.04 and 0.26 to 0.3. Embryo survival occurred at 0.05 to 0.25 ratios. At viral load of 100EID_50_, there was no embryo survival at ratios of 0.01 to 0.14 and 0.21 to 0.3; embryo survival occurred at ratio of 0.15 to 0.2. The effect of changing Zn to TMP ratio on embryo survival is shown in Figs. 2 and 3. The effect of increasing viral load on embryo survival is shown in Fig. 4.

It appeared that with increasing the ratio of Zn to TMP embryo survival increased. It peaked at ratio about 0.18 (Zn/TMP). There was decrease in the survival with further increase in the ratio. The data were normally distributed. The changes in Zn to TMP ratio had significant effect on the survival of the embryos, p < 0.001. In cluster analysis, it seemed that there were three clusters, with one cluster showing much better survival than the other two clusters, p < 0.001. In that cluster, the range of effective ratio was 0.1 to 0.2. The peak of surviving embryos was at ratio of 0.18, Fig. 2. With increasing viral load to 100 EID_50_, the cluster distribution was abolished. Embryo survival was possible in Zn/TMP ratio range 0.15–0.2, with the peak survival at ratio of 0.18, Fig. 3.

In C1, C2 and C3 groups, all embryos died. In C4, embryos survived which confirms that death of the embryos was a result of influenza virus infection.

**Fig. 5:**
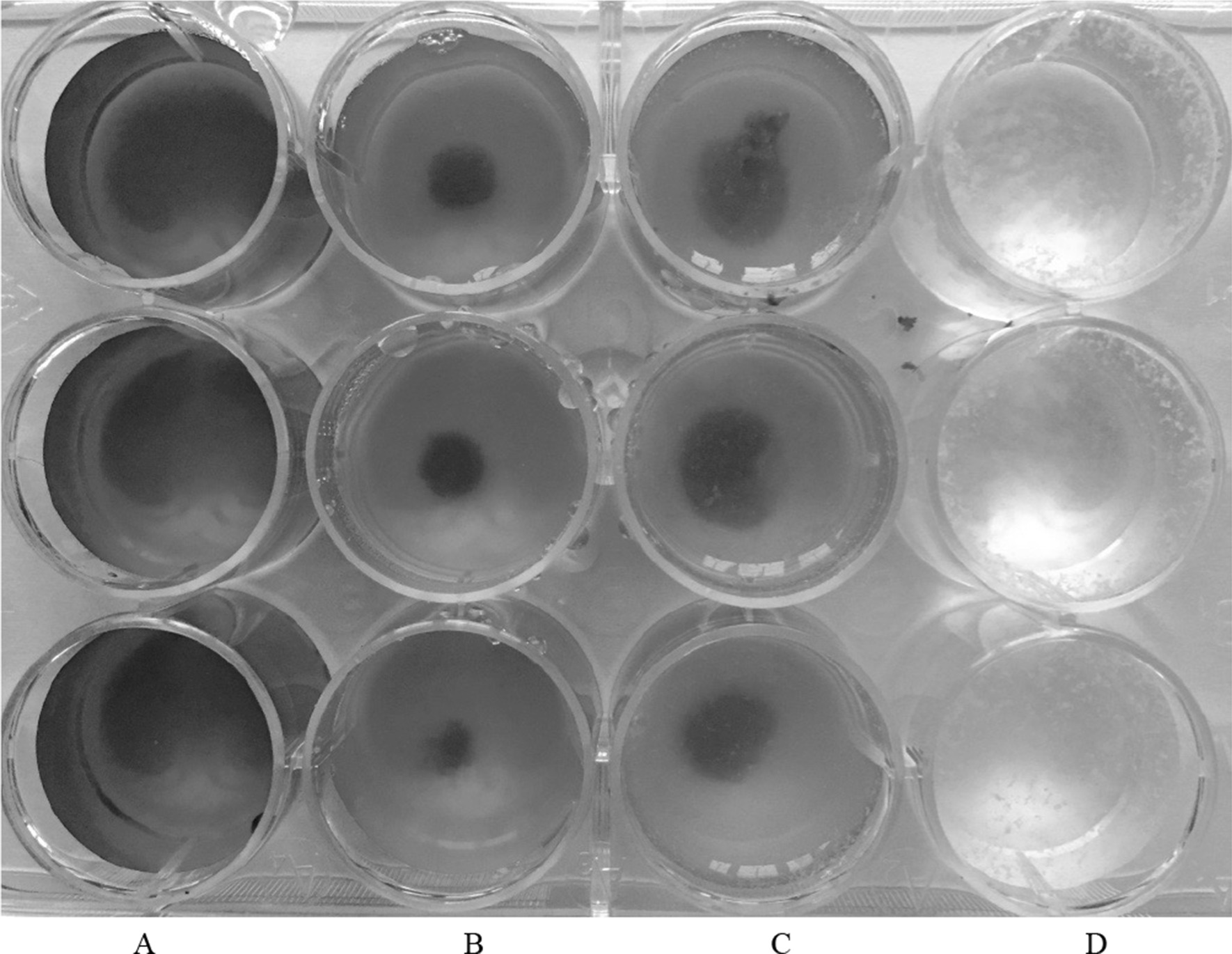
It shows haemagglutination inhibition test using chicken red blood cells. In A, convalescent serum was added to neutralise the virus, there was no agglutination (−). In B, Tri-Z was added at 5 mcq/ml, haemagglutination occurred but to less extent (++) than that of no treatment in C (++++). In D, no red cells were added

The results from HAI studies whereby Tri-Z was mixed with the virus prior to addition RBCs and that where Tri-Z was incubated with RBCs prior to addition to the virus were the same. The highest dilution of the virus stock that caused HA was 1/4096, thus containing 1HAU. The dilution used that contained 4 HAU was 1/1048. It was possible to achieve total HAI (score of 0) with Tri-Z at 15 µg/ml. However, at 5 µg/ml, HAI was partial scoring ++ comparing to controls of virus scoring ++++ and without virus scoring 0. Neither Zn nor TMP separately had any observable HAI effect (scoring +++) similar to control virus, Table 2 and Fig. 5.

**Table 2:** It shows that Tri-Z, at ratio 5 mcq/ml, had hem-agglutination inhibition effect scoring ++ comparing to TMP or Zn separately; − means no hem-agglutination, ++++ mean total hem-agglutination

Agent	Hemagglutination
saline	−
virus	++++
serum and virus	−
Tri-Z and virus	++
TMP and virus	++++
Zn and virus	++++

Serum preparation from patients and hemagglutination were carried out as previously described [23, 24]

## Discussion

This study shows that a combination of TMP and Zn (Tri-Z) can treat influenza virus infection in chick embryo independent of interferon. Six days old chick embryos treated with Tri-Z survived the virus, comparing to controls. In 6 day old chick embryo, interferon is deficient [26] and the viral infection is lethal. Using TMP, zinc or normal saline separately did not enable embryo survival of the infection at 10 EID_50_ or 100 EID_50_. However, neutralizing antibodies in convalescent serum enabled embryos to survive which confirmed that the cause of its death was infection by the virus.

Like other RNA viruses, influenza virus depends on its host cell and nuclear DNA for replication and spread [3], which is associated with an increase in amino acid synthesis [29]. Excluding host nuclear DNA by enucleation of cells prevents RNA virus replication [30, 31]. Interfering with host DNA by TMP (which is a dihydro-folate reductase enzyme inhibitor) can disturb the hots processes for viral replication. While in bacteria, TMP affects DNA to a point that causes arrest of cell division (bacteriostasis) in man the effect is transient [6]. It was reported that TMP may have beneficial effect in treating DNA virus infections [32, 33]. However, for RNA virus, TMP on it is own is not effective. All embryos treated with TMP alone did not survive the infection, but with Zn, the combination was effective.

Zinc was shown to inhibit replication of RNA virus, rhinovirus, in vitro [7]. In vivo, the effect was not quiet evident but thought to assist in ameliorating rhinovirus infections (common cold) [34]. In chick embryo, zinc did not inhibit influenza virus. All embryos treated with Zn alone died from influenza virus infection. But, with TMP, the combination enabled embryo survival of influenza virus.

Zinc ions are key structural components of a large number of proteins. In eukaryotic cell under optimal conditions, Zn associates with protein forming Zn finger. A Zn finger is a small protein structural motif, characterized by the co-ordination of one or more zinc ions in order to stabilize the protein fold. Zn fingers bind RNA and mediate protein–protein interactions. A Zn finger protein was found to inhibit influenza virus replication [35–38]. Under experimental conditions, Zn combines with TMP forming fixed complex preserving the anti-bacterial properties of TMP but without evidence of anti-virus [39, 40]. Whereas, in-vivo, the non-fixed formulation of Zn and TMP (Tri-Z) inhibited influenza virus in chick embryo, similar to that in man [8], enabling embryo survival of infection. The viral inhibition of Tri-Z appears to be ratio dependent. We found that increasing ratio of Zn to TMP increased embryo survival, until an optimal ratio was reached, then embryo survival decreased with further increase in ratio. Increasing viral load from 10 EID_50_ to 100 EID_50_ reduced the range of the ratio that enables embryo survival. However, the optimal ratio remained unchanged. The ratio dependence indicates presence of an interaction between the two molecules when certain conditions are met. For example, in water, the pH of the combination at the optimal ratio of 0.18 is 7.24 which is the pKa of TMP. For TMP, pH = pKa + Log10 (protonated/unprotonated compound), as pH = pKa, then the ratio of protonated to unprotonated TMP is 1. Such equilibrium in the charge of TMP is expected to allow association with Zn in a dynamic combination. Dynamic interactions between drugs were shown to occur at equilibrium and enhance its permeability across biological membrane to target RNA [41, 42]. This, perhaps, is similar to the Zn finger protein phenomenon.

Once introduced, the virus is not shed out of the egg. Inability of the virus to infect suggested that Tri-Z prevented these infections from occurring. A possible mechanism of action is interfering with virus binding to its cell surface sialic acid-based receptor [43]. Virus binding to this receptor is a process by which HA occurs [23]. At ratio 0.18 Zn to TMP, hemagglutination inhibition was possible. Previous studies showed a relationship between pharmacokinetics of TMP and sialic acid rich protein [44, 45]. Therefore, it seems that Tri-Z acts at multiple sites whereby the RNA virus utilizes the host to infect and replicate including the virus ability to bind to its cell surface receptor.

It may be thought that increasing use of TMP in this combination might increase bacterial resistance. TMP is a long-standing drug which is widely used and spread in the environment without emergence of new resistance [46].

## Conclusions

We conclude that a combination of Zn and TMP enables chick embryo survival of influenza virus infection which is ratio dependent. At optimal ratio, the combination acts on host cells including cell surface receptor whereby it has haemagglutinin inhibition effect. Further studies to examine the use of Tri-Z in treating patients infected with other respiratory RNA viruses are planned.

## Data Availability

All data are presented in the results section and expressed into graphic presentations. If any data, not already shared, are needed it may be made available.

## Abbreviations

TMP: Trimethoprim
Tri-Z: Trimethoprim and zinc combination
Zn: Zinc
HA: Hemagglutination
HAI: Hemagglutination inhibition
HAU: Hemagglutination unit
ml: Milliliter
RBC: Red blood cells
EID: Egg infective dose
^o^C: Degree centigrade
WHO: World Health Organization
FIA: Florescence immune assay
RT-PCR: Reverse transcription polymerase chain reaction
RNA: Ribonucleic acid
DNA: Deoxyribonucleic acid
PBS: Phosphate buffered saline
